# Non-Coding RNAs as Blood-Based Biomarkers in Cardiovascular Disease

**DOI:** 10.3390/ijms21239285

**Published:** 2020-12-05

**Authors:** Raquel Figuinha Videira, Paula A. da Costa Martins, Inês Falcão-Pires

**Affiliations:** 1 CARIM School for Cardiovascular Diseases, Faculty of Health, Medicine and Life Sciences, Maastricht University, 6229 ER Maastricht, The Netherlands; r.figuinhavideira@maastrichtuniversity.nl (R.F.V.); p.dacostamartins@maastrichtuniversity.nl (P.A.d.C.M.); 2 Department of Molecular Genetics, Faculty of Science and Engineering, Maastricht University, 6229 ER Maastricht, The Netherlands; 3 Cardiovascular Research and Development Center, Faculty of Medicine, University of Porto, 4200-319 Porto, Portugal

**Keywords:** ncRNAs, biomarkers, cardiovascular diseases, diagnosis

## Abstract

In 2020, cardiovascular diseases (CVDs) remain a leading cause of mortality and morbidity, contributing to the burden of the already overloaded health system. Late or incorrect diagnosis of patients with CVDs compromises treatment efficiency and patient’s outcome. Diagnosis of CVDs could be facilitated by detection of blood-based biomarkers that reliably reflect the current condition of the heart. In the last decade, non-coding RNAs (ncRNAs) present on human biofluids including serum, plasma, and blood have been reported as potential biomarkers for CVDs. This paper reviews recent studies that focus on the use of ncRNAs as biomarkers of CVDs.

## 1. Introduction

Cardiovascular diseases (CVDs) including aortic stenosis, hypertension, myocardial infarction, congenital heart diseases, aortic aneurysms, and right ventricle dysfunction can lead to heart failure (HF) and, ultimately, death. CVDs alone are responsible for more than 17.9 million deaths per year, corresponding to 31% of all deaths globally and being the first cause of morbidity and mortality worldwide (World Health Organization (WHO), data from 2019 [1]). Unfortunately, for most CVDs, treatment efficacy and outcome remain highly compromised by incorrect or late diagnosis. This unpredictability is attributed to the scarcity of obvious symptoms that indicate cardiac dysfunction, thus some CVDs are classified as “silent killers”, contributing to a diagnosis that occurs after acute and/or severe symptoms episodes, usually occurring at a stage where reverting cardiac damage is no longer possible (WHO [1]).

Clinical management of CVDs could be facilitated by detection of blood-based biomarkers that reliably reflect the current condition of the heart. The term biomarker arises from the junction of the words “biological” and “marker”. Biomarkers are defined by WHO as “any substance, structure, or process that can be measured in the body or its products and influence or predict the incidence of outcome or disease”. Later, the definition of a biomarker was extended to “any measurement reflecting an interaction between a biological system and a potential hazard, which may be chemical, physical, or biological” [2].

Ideally, a human biomarker should be of easy access and acquirement, display a high degree of specificity and sensitivity, be stable in its environment (plasma, urine, blood, and saliva, among others), and thus have little or no variability [3]. While it is desirable that a biomarker can be determined by a simple, fast, and low-cost method [3], very few molecules satisfy these criteria and rather just meet a reduced number of these requirements. Up to date, CVD biomarkers are restricted to troponins, natriuretic peptides (namely, atrial natriuretic peptide (ANP) and brain natriuretic peptide (BNP)), matrix metalloproteases, and galectin-3, but other promising factors, such as non-coding RNAs, are slowly being introduced as both prognostic and diagnostic tools in cardiovascular clinical practice [4]. The search for ideal CVD biomarkers has deepened in the past years; however, there is still a long way to go until the golden biomarkers enter clinical practice.

In the recent years, a new class of potential biomarkers, including non-coding RNAs (ncRNAs), has received much attention in the cardiovascular field. Representing almost 60% of cell transcriptome, ncRNAs are functional RNA molecules that, despite lacking a protein-coding region, are essential players in gene regulation and, consequently, in cell function and survival [5]. The discovery of the first ncRNA lin-4, a small RNA able to decrease the translation of another gene, *Lin-14*, by directly binding to lin-14 RNA molecule [6], opened doors to discover many other forms of ncRNAs including microRNAs (miRNAs, miRs), long non-coding RNAs (lncRNAs), and circular RNAs (circRNAs), among others.

MiRNAs are small ncRNA (18–25 nucleotides) that inhibit the expression of their target gene(s) by sequence-specific recognition. The transcript-binding sequence is usually located in the 3′-untranslated region (3’-UTR), whereas the complementary seed sequence is located in the first two to eight nucleotides of the miRNA [7]. The degree of binding complementary between miRNA and mRNA sets the outcome of target transcript expression with high complementary, leading to transcript destabilization followed by degradation in processing bodies (p-bodies) [8]. Meanwhile, a low complementary degree leads to translational repression, as binding to the mRNA cap prevents the translation initiation factor eIF4E, inhibiting the assembly of the ribosomal subunits or even promoting premature ribosomal drop-off and mRNA release from the ribosomal translational complex [9,10,11]. All of these regulatory mechanisms potentially enable a single miRNA to control hundreds of different target transcripts. Besides these endogenous mechanisms of action, cells can also secrete miRNAs that will be found extracellularly in human biofluids such as saliva, urine, serum, and plasma [12].

Although miRNAs are established as an important and possibly the most described group of ncRNAs, the largest portion of the ncRNA transcriptome is composed of lncRNAs. LncRNAs are a class of non-coding linear transcripts of more than 200 nucleotides in length, which frequently miss an open reading frame (ORF) and influence gene expression in a variety of manners [13]. Notwithstanding, some lncRNAs may display protein-coding functions, and several shared attributes of mRNAs such as 5′ cap, more than one exon, alternative splicing, and poly(A) tails [13]. LncRNAs are commonly classified according to their genomic localization; those entirely transcribed from intronic regions of protein-coding RNA are termed intronic lncRNA, whereas the case wherein a lncRNA is transcribed from an intergenic region is named intergenic lncRNA. Additionally, a lncRNA could also be transcribed from an enhancer region, a regulatory DNA sequence of gene expression that increases promoter’s activity, which are named eRNAs [14].

Similar to miRNAs, lncRNAs can also be found in different cellular compartments such as nucleus, cytoplasm, and extracellular space. In the nucleus, lncRNAs will influence transcription by recruiting regulatory factors and catalytic proteins; alternately, eRNAs drive proteins to enhancer regions, anchoring them to DNA in the right position and organizing chromatin interaction [15]. Oppositely, some lncRNAs recruit polycomb repressive complex 1 and 2 (PRC1, PRC2), promoting methylation marks and decreasing transcription [16,17]. In the cytoplasma, lncRNAs may act as miRNA sponges, preventing small ncRNAs from binding to their target transcripts or, alternatively, lncRNAs can serve as scaffolds for nucleoprotein complexes influencing mRNA translation and interfering with mRNA splicing and degradation [18,19]. Despite their many described functions, lncRNAs are still an understudied class, with many lncRNAs remaining to be identified and their functions described. Similar to miRNAs, lncRNAs can also be found in extracellular spaces and fluids, encapsulated in vesicles or bound to other particles, and have, therefore, also been studied as potential biomarkers of CVDs. Unlike protein-coding and other ncRNAs, lncRNAs sequence is poorly conserved among species, despite that the promoter regions of lncRNAs are often conserved, as well as their structure (namely their 3D conformation and shape) and their functionality [13].

A newly found class of ncRNAs is circRNAs, which, in contrast with previously reported ncRNAs, are characterized by a circular form that arises as a consequence of back splicing events [20]. CircRNAs are small, single-stranded RNA sequences with loop structures covalently closed that confer them, unlike linear ncRNAs, protection from degradation by ribonucleases [20]. CircRNAs can act as decoy elements working through protein interaction; regulate mRNA splicing processes by recruiting splicing factors; and, under special conditions, they could be translated into proteins once internal ribosome entry site (IRES) was initiated [21].

Furthermore, circRNAs are conserved among species, with some of them displaying cell-type and spatial-temporal specificity [22]. Emerging evidence suggests a myriad of mechanisms by which circRNAs can influence gene expression, participating in cell function and contributing to pathological processes, including several ones associated with CVDs [23]. Another peculiarity of circRNAs is the presence of multiple miRNA response elements (MERs), which may compete with miRNA targets for binding and, as such, serve as miRNA sponges [23].

CircRNAs can also affect gene transcription of the so-called parent gene, the gene that mRNA gives origin to the circRNA after back splicing, through interaction with RNA polymerase II and regulation of its transcriptional activity [24]. As for other ncRNAs, circRNAs can also be detected in different human biofluids, and their role as biomarkers has been recently reported in cancer [25,26].

Here, we will review the literature on the detection of ncRNAs in human samples of blood, plasma, and serum and conclude on their potential as biomarkers of CVDs.

A variety of cardiac diseases have different etiologies and specific events that can lead to the expression and circulation of cardiac-derived biomarkers

## 2. Aortic-Related Diseases

### 2.1. Aortic Valve Stenosis

Aortic valve stenosis (AS) affects more than 5% of the population above 65 years old. It is defined as a narrowing of the aortic valve that progressively imposes resistance to the blood flow from the left ventricle (LV) to the aorta [27]. Unfortunately, as AS symptoms occur at an advanced disease stage, where cardiac damage is no longer reversible, the disease has a dismal prognosis in symptomatic individuals. Currently, there is no pharmacological treatment for AS and the most efficient solutions include surgical aortic valve replacement (SAVR) or trans-catheter aortic valve replacement (TAVR). However, valve replacement has been associated with risk of vascular access-site bleeding, blood transfusion-related infections, stroke, para-valvular leaks, and heart block, which also increase the risk of HF and death following valve replacement [28].

ncRNAs have been studied as prospective biomarkers of AS thanks to their potential to reflect AS pathogenesis and to be detected at an earlier AS stage. Blood-based ncRNA biomarkers could aid in patient’s risk stratification and decision making, such as the timing and type of intervention (SAVR vs. TAVR), as well as in predicting AS progression.

Clinically, AS is associated with LV hypertrophy and cardiac fibrosis. In fact, a study measuring the plasma levels of miR-1, miR-133, and miR-378 in AS patients suggested that all three miRNAs were downregulated [29]. Yet, miR-378 is even lower expressed in patients with LV hypertrophy in comparison with those without hypertrophy, which indicates a strong negative correlation between miR-378 and LV mass and placing miR-378 as an independent predictor of LV hypertrophy in AS [29]. In a similar cohort of AS patients, circulating miR-210 was shown to be increased and to inversely correlate with LV end diastolic dimensions, cardiac parameters affected in AS [30]. This miRNA may also predict mortality as higher levels of miR-210 were found in patients with a high risk of mortality in follow-up studies. Although miR-210 is not established as an AS-specific marker, the results reported are comparable to data obtained for the N-Terminal fragment of BNP levels and, when combined with other cardiovascular (CV) parameters, could help predict CV risk associated with AS [30].

Another study showed the presence of myocardial fibrosis in severe AS patients with LV preserved ejection fraction (EF). Interestingly, fibrosis directly associates with miR-21 plasma levels, suggesting that miR-21 levels reflect the degree of myocardial fibrosis [31]. Furthermore, plasma miR-21 levels provided more accuracy, sensitivity, and specificity to distinguish myocardial fibrosis when compared with common AS parameters such as global longitudinal strain and BNP levels [31]. Similar results were found by Villar et al., unravelling that not only is circulating miR-21 increased in AS patients compared with healthy controls, but also an increase in circulating miR-21 increase is proportional to an increment in myocardial miR-21 expression as well as of cardiac fibrotic genes [32].

Analysis of peripheral blood collected from AS patients demonstrated that serum miR-19b levels are abnormally decreased and inversely correlated with LV stiffness and collagen cross-linking when matched to age and gender healthy controls [33]. Previously, members of the miR-17-92 cluster, to which miR-19 belongs, were reported to regulate myocardial fibrosis and angiogenesis [34]. However, the role of angiogenesis is still unclear, with some studies reporting that the degree of myocardial angiogenesis is accompanied by increased hypertrophy, worsening of systolic function, and severe AS [35,36]. On the other hand, a study demonstrated that increased angiogenesis and cardiomyocytes proliferation prevent maladaptive remodeling in a model of LV pressure-overload [37].

Interestingly, a number of different miRNAs have been related to different phenotypes of AS [38]. For example, AS patients with a low flow condition display high levels of miR-1, miR-21, and miR-133, whereas higher levels of miR-133 reflect LV hypertrophy [38]. In fact, patients with severe AS and reduced EF demonstrated increased levels of miR-1, miR-29, and miR-133 [38]. Curiously, in conditions of pressure-overload such as AS, miR-29 levels were clinically different among males and females [39]. Above the age of 50 and compared with control healthy women, women with AS showed a significant enrichment of miR-29 that is associated with increased LV mass and concentricity [39].

Despite certain circRNAs showing aortic valvular tissue specificity, and several associations that have been suggested between lncRNAs and AS pathology, to date, no studies have reported the association of AS with either plasma circRNAs or lncRNAs [40,41].

### 2.2. Aortic Aneurysm

Aortic aneurysm (AA) represents an important cause of death. AA is clinically characterized by an enlargement of at least 50% of an aortic segment compared with the same segment in healthy individuals and can be subdivided in thoracic aortic aneurysm (TAA) or abdominal aortic aneurysm (AAA), the latter being the most frequent [42]. AA is accompanied by progressive degeneration of the aortic wall [42]. Initially, a bulge-like dilation of a segment of the aortic wall tends to expand and increase the risk of rupture [42].

AA incidence and prognosis is associated with patient’s sex and age, typically men and elderly populations are more affected by AA and demonstrate worse outcomes [42]. To date, there are no efficient pharmacological therapies against AA; therefore, when an AA progresses to a severe stage, surgery procedures are required [42].

An early and correct diagnosis could avoid surgery and improve patient’s outcome. As nearly all patients are asymptomatic, an early diagnosis becomes difficult [43]. Most of the cases are detected during screenings using imaging techniques such as computerized tomography angiogram (CTA), ultrasound sonography (US), and magnetic resonance imaging (MRI) [42]. However, these techniques are expensive and incur hazards, thus new markers are needed, which encourages the exploration of ncRNAs’ potential candidates. Recently, blood-based biomarkers such as tenascin-C, C-reactive protein, cystatin C, cathepsin, iron, immune system cells (lymphocytes and monocytes), genetic markers, and ncRNAs have emerged as attractive alternatives to help in AA diagnosis [44].

Among ncRNAs, miRs continue in the front of the biomarkers run as the most well described and studied ncRNAs. Therefore, it is not surprising that most AA related studies on ncRNAs focus on miRs.

In 2020, a small study described seven circulating miRs with an altered expression in AAA condition (*n* = 16) when compared with the control (miR-103a-3p, miR-27b-3p, miR-99a-5p, miR-375, miR-221-3p, miR-146a-5p, and miR-1260) [45]. After variable adjustment, only miR-221-3p and miR-27b-3p remained significantly overexpressed in AAA plasma, suggesting its potential as AAA biomarkers [45].

A work from *Wanhainen* et al. analyzed the circulating miRNA profile of 169 AAA patients and contrasted it against the profile of 48 healthy age and sex-matched individuals [46]. Of a predefined panel composed of the 172 most expressed miRs in plasma, 103 were found to be differentially expressed in the plasma samples of AAA patients relative to the plasma samples of healthy individuals [46]. The top altered miRs included miR-10b-5p, which displayed a specificity of 70% and a sensitivity of 60%, but, by adding let-7i-5p to the analysis, a specificity of 71% at a sensitivity of 90% was reached, allowing the discrimination between controls and AAA patients [46]. Despite improved sensitivity after combining different miRs, this result is still disappointing when compared with other studies and biomarkers.

On the other hand, lncRNAs were also reported as potential biomarkers for TAA [47]. In fact, TAA patients revealed decreased LUCAT1 and SMILR plasma levels when compared with a control group [47]. However, only LUCAT1 demonstrated an area under curve (AUC) higher than 0.65, indicating a modest potential diagnostic value for TAA [47].

More promising results were obtained by Tian et al., who investigated the potential role of plasma circMARK3 to identify an advanced stage of aortic aneurysms, such as aortic dissection (when the inner aortic wall becomes tore), particularly acute aortic dissection -Standford type A (AAAD) [48]. The results showed that 506 circulating circRNAs were significantly dysregulated in AAAD cohort group compared with controls, including circRNAs that were 320 were significantly increased and 186 circRNAs that were significantly decreased [48]. From these, circMARK3 was chosen for further validation as a biomarker because of its high expression on AAAD [48]. After receiver operating characteristic (ROC) analysis, serum circMARK3 was characterized by an AUC of 0.9344 (using a cutoff value of 1.497), a sensitivity of 90.0%, and specificity of 86.7% [48]. Further combination of serum circMARK3 and miR-1273-3p revealed even improved results, namely, sensitivity and specificity were increased to 93.3% and 86.7%, respectively. The AUC of the combined ncRNAs was 0.9644 when using a cutoff value of 0.4807 [48].

Together, the results obtained by Tian et al. are very encouraging and highly suggest serum circMARK3 and miR-1273-3p as potential biomarkers for the AAAD condition.

## 3. Coronary Artery Disease

Often viewed as an inflammatory disorder, coronary artery disease (CAD) occurs when vessels that supply blood to the heart (coronary arteries) become damaged [49]. Artery plaque buildup due to atherosclerosis is a frequent cause of CAD, resulting in the narrowing of coronary arteries followed by myocardial ischemia and, ultimately, thrombosis and HF [49]. As the presence of plaques is influenced by age, smoking, and an unhealthy lifestyle, CAD affects mainly adults and elderly populations, displaying worse prognosis in developing countries [49]. Current therapeutic strategies are focused on pharmacological interventions to reduce the risk of atherosclerotic complications (decrease low density lipoprotein (LDL) levels, anti-thrombotic drugs) and symptoms (beta blockers and ranolazile, a drug that prevents late phase of the inward sodium current contributing to cardiac relaxation during diastole) [49]. At an advanced disease stage, mechanical interventions such as angioplasty, percutaneous coronary intervention (PCI) or coronary bypass surgery are needed to vascularize the blocked artery and to re-establish blood flow [49]. Although early CAD diagnosis is imperative to prevent plaque rupture and further associated complications, the existing methods, such as computed tomography, electrocardiogram (ECG), and echocardiography, may not be applied to the broad population owing to limited equipment availability, operated-dependent results, time constraints, and costs.

As CAD pathology is directly influenced by endothelial dysfunction, is it feasible to assume that endothelial-related miRNAs could constitute reliable biomarkers of CAD. Accordingly, miR-17 described as a negative regulator of tumor angiogenesis is highly expressed in plasma from patients with severe CAD and is a potential biomarker candidate [50]. Other miRNAs have been portrayed as potential biomarkers in plasma of CAD patients when compared with healthy control groups. Such is the case of plasma increased levels of miR-33, miR-208b, and miR-499, as well as decreased expression of miR-155, miR-145, and let-7c [51,52,53].

Notwithstanding, the levels of circulating miRNAs are not only capable of indicating the presence of CAD lesion, but also can differentiate and correlate with CAD severity, as shown for miR-206 [54]. Patients with major blocked coronary arteries or with a higher number of blocked coronary arteries have higher levels of miR-206 when compared with patients with less severe lesions or healthy controls [54]. As such, miR-206 is upregulated in individuals with three blocked coronary arteries when compared with individuals two blocked coronary arteries, and those showed enhanced levels of miR-206 when compared with individuals with only one blocked coronary artery.

Analysis of atherosclerosis- and cardiac-related lncRNAs levels in peripheral blood mononuclear cells (PBMCs) from CAD patients and healthy individuals revealed that three lncRNAs, KCNQ1OT1, HIF1A-AS2, and APO1, are significantly increased in patients when compared with the healthy controls [55]. Of the three, APO1 revealed the best diagnostic value with a sensitivity of 100% and a specificity of 80% [55]. The combination of these three lncRNAs improved the diagnostic score by increasing the specificity to 90% and maintaining the sensitivity at 100%. These results place KCNQ1OT1, HIF1A-AS2, and APO1 at the top of potential CAD biomarkers to be used in the clinics [55]. Another study revealed HOTAIR, an lncRNA reported to be involved in vascular inflammation and age-associated-CVDs, as being upregulated in both plasma and PBMCs samples of CAD patients when compared with non-CAD patients [56]. Assessment of lncRNA expression profiles in plasma samples of both groups detected several differentially expressed lncRNAs, including lncRNAs GAS5 [57]. Although previously associated with several CVDs such as diabetes mellitus, hypertension, and valvular disease, a recent study has reported GAS5 to be significantly downregulated in CAD and diabetes mellitus, but not altered in other cardiovascular diseases such as hypertension, abnormal aortic aneurysm, viral myocarditis, atrial fibrillation, valvular disease, dilated cardiomyopathy, and peripheral artery disease [57].

A similar approach was taken by *Vilades* and colleagues, who categorize CAD patients according to the levels of plasma circ_0001445 (circSMARCA5), whose expression inversely correlated to coronary atherosclerosis extension and severity [58]. In fact, augmented circSMARCA5 levels are associated with a decreased segment stenosis score as well as with decreased cardiovascular risk [58]. Furthermore, RNA sequencing from plasma exosomes of CAD and non-CAD patients identified 335 exosomal circRNAs to be differentially expressed among the two groups [59]. After adjusting for risk factors, circ0005540 was upregulated in CAD patients, and displayed high sensitivity and specificity for identifying CAD patients, suggesting its potential as a CAD biomarker [59]. Similarly, circZNF609 also seems to be a promising biomarker for CAD [60]. CirZNF609 expression in peripheral blood leucocytes is decreased in CAD patients when compared with the control cohort [60]. Despite the different etiologies of CAD, overall, circZNF609 featured a specificity of 0.765 (76.5%) and a sensitivity of 0.804 (80.4%), indicating a moderate predicting value to identify CAD patients [60]. Furthermore, a microarray analysis of CAD and control PBMCs detected upregulation of circ_0001879 and circ_0004104 in CAD patients and these levels were associated not only with standard CAD biomarkers, but also with CAD risk factors [61]. Whereas both circRNAs can be individually used as biomarkers, combining both only showed an improved diagnostic value when combined with conventional CVD markers such as serum creatinine and CVD risk factors as hypertension, high LDL, smoking, and drinking, among others [61].

## 4. Myocardial Infarction

One of the consequences of CAD is the formation of a blood clot that, among other causes, occludes coronary arteries, resulting in ischemia and myocardial infarction (MI, also known as heart attack). In fact, a correlation between CAD and MI was previously established, namely on how well plasma miRNAs can predict CAD progression towards acute MI. A higher number of stenosed coronary vessels is associated with lower plasma miR-99a levels in MI patients [62]. Following PCI, miR-99 expression levels were re-established to comparable levels as observed in healthy volunteers. In MI, lower plasma miR-99 levels negatively correlated with those of cardiac troponin I and creatinine kinase (markers of cardiac dysfunction), suggesting that miR-99 expression might be necessary for proper cardiac function [62]. Similarly, miR-181a was also suggested to be a biomarker for MI as its increased plasma concentration is proportional to the severity of coronary stenotic lesions as well as deterioration of LV function [63]. Accordingly, miR-181a levels were positively correlated with creatinine kinase levels, and reduced 48 h after PCI [62,63].

Data from Centers for Disease Control and Prevention (CDC), a U.S. national public health institute, respective to the U.S. population, reported that, every 40 s, someone dies from a heart attack, and for every five occurrences, one is silent, meaning that no symptoms are recognized by the patient. Commonly, MI is diagnosed by combining an ECG test and analysis of biomarkers such as cardiac troponin I and T levels. However, ECG can only detect 30–70% of MI cases, highlighting the need for more accurate and sensitive biomarkers [64].

Various plasma miRNAs have been described as potential indicators of myocardial infarction, likely the most studied and described event among all cardiovascular diseases. Hence, miR-1, miR-126, miR-30a, miR-195, miR-26a-1, miR-146a, and miR-199a-1 were revealed to be upregulated in individuals diagnosed with acute MI (AMI) [65,66,67], with some of them, that is, miR-1, miR-126, miR-30a, and miR-195, reaching their maximal expression 8 h after the onset of symptoms [65,67,68]. In contrast, let-7b and miR-132-5p levels were decreased in acute MI patients when compared with control subjects [65]. AUC of ROC analysis only granted a modest individual value of sensitivity and specificity (below 90%), suggesting that each miRNA has moderate potential in the diagnosis of AMI patients. However, clustering of different miRNAs was more encouraging as an AUC of 0.913 was obtained when grouping miR-26a-1, miR-146a, and miR-199a-1 [66]. Other miRNAs such as miR-22-5p and miR-150-3p were also increased in MI by 36.47- and 4.09-fold, respectively, and even though they continued to be elevated for 72 h, the peak was reached at the onset of symptoms [69]. The sensitivity and specificity of the different miRNAs were calculated at different time points. Despite the fact that individual miRNAs showed a moderate power, when combined together, miR-132-5p, miR-22-5p, and miR-150-3p presented a better diagnostic value to distinguish AMI patients [69].

Microarray analysis in plasma samples of a small group of AMI patients identified 33 differentially expressed miRNAs, from which miR-30d-5p and miR-125-5p were selected for validation in a larger study population composed of 230 AMI patients and 79 healthy controls [70]. Despite the notable diagnostic value (namely specificity and sensitivity) of cardiac troponin I, the results obtained for miR-30d-5p surpassed the performance of cardiac troponin I by displaying an AUC of 0.915 [70] and highlighting the potential diagnostic use of miR-30d-5p and miR-125-5p in patients suspected of developing AMI [70].

Less promising results were reported for miR-221-3p, which, despite being upregulated by 3.89-fold in AMI [71], and an AUC of 0.881, did not reach the values of the standard marker troponin and neither improved the AUC levels when combined with troponin [71]. In line, miR-486 and miR-150 were also described as “exhibiting strong differentiation power” between healthy controls and AMI patients. However, the combination of both miR-486 and miR-150 only showed a moderated predictive power with an AUC of 0.731 [72]. Nevertheless, these two miRNAs were able to modestly classify AMI patients into ST elevation MI (STEMI) and non-ST elevation MI (NSTEMI) [72]. Typically, STEMI is associated with a more severe condition and worse prognosis.

Interestingly, even after pharmacological treatment, progression towards maladaptive remodeling occurs in approximately 30% of AMI patients [73]. A total of 14 plasma miRNAs and 16 serum miRNAs were found to be dysregulated in samples from AMI subjects [74]. Following cross data and validation (in different cohorts), only miR-30a-5p was significantly upregulated in patients with (HF) after an MI episode. Interestingly, miR-30a-5p levels at the onset of AMI negatively correlate with LVEF six months after AMI, suggesting a prognostic value for miR-30a-5p, despite that it might also represent a marker of myocardial ischemia. The discriminatory power of miR-30a-5p to identified HF versus non-HF patients after MI was assessed by AUC calculation, resulting in an AUC of 75% [74].

As mentioned previously, one mechanism of action of lncRNAs is by competitively binding to miRNAs’ target binding site, which prevents the attachment of mRNA–miRNA, thus inhibiting miRNA action. For example, lncRNA HOTAIR has a sponge like effect on miR-1, an miRNA upregulated in AMI cases [75]. HOTAIR is decreased in serum from subjects with AMI, reaching its lower expression 6 to 12 h after the onset of the first symptoms and negatively correlating with its target, miR-1 and cardiac troponin I [75]. Three days after the AMI episode, miR-1, cardiac troponin I, and HOTAIR levels are re-established [75]. Additionally, a study in mice subjected to hypoxia demonstrated that HOTAIR is myocardium-specific and that plasma HOTAIR in mice behaves similar to what was observed in humans, suggesting its myocardial origin [75].

Another myocardial enriched lncRNA is UCA1, which, similar to HOTAIR, is also decreased in the plasma of AMI individuals [76]. By clustering AMI patients according to the time from initial symptoms, it was possible to analyze the time course expression of UCA1 following an MI event [76]. While the lowest levels were reached between 6 and 12 h and remained low up to 48 h after AMI, from this point on, UCA1 levels increased and, at 96 h post-AMI, they were higher in AMI patients compared with the controls [76]. Interestingly, UCA1 also negatively correlates with miR-1 expression, but no mechanism has yet been described associating the two ncRNAs [76]. Although the predictive value of UCA1 alone is not as potent as standard biomarkers, the overall predictive power of UCA1 for AMI was increased to 0.983 AUC when combined with creatinine kinase [76].

Other lncRNAs have been attributed biomarker potential when screening for differentially expressed lncRNAs in plasma of 46 STEMI patients. While aHIF, KCNQ1OT1, and LIPCAR were found to be increased, six others, including HOTAIR, UCA1, MIAT, MALAT1, ANRIL, and CPNE3, were decreased in these patients [77]. The most promising diagnostic values were revealed for LIPCAR, with a sensitivity of 82% and a specificity of 75% in distinguishing STEMI subjects, and with LIPCAR levels positively correlating with cardiac troponin I and creatinine kinase and inversely with LV ejection fraction [77]. Notably, the increased levels of LIPCAR observed upon an STEMI event were decreased shortly after PCI [77].

Despite the relatively new role of circRNAs as biomarkers of AMI, Deng et al. found 160 circRNAs to be differentially expressed in AMI patients, with 87 of them being increased and 73 decreased when compared with healthy controls [78]. Among the most downregulated was circRNA_081881, showing a 12.5-fold change compared with the control group [78]. Currently, the most studied circRNA in MI is the myocardial infarction-associated circular RNA (MICRA) [79,80], which, by being decreased in plasma of MI patients, positively correlates with LV ejection fraction and accentuates the risk of LV dysfunction [79].

## 5. Congenital Heart Defects

In the past decade, prenatal testing made crucial advances and increased the survival rate up to 87% in the first year. Currently, neonatal congenital heart defects (CHDs) have an incidence of 4–8 per 1000 births and their manifestations range from minor defects (such as small intracavitary communications) to severe defects that can even lead to prenatal lethality (CDC data, September 2020). The most common include tetralogy of fallot (TOF), atrial septal defect (ASD), ventricular septal defect (VSD) single ventricle (SV), persistent truncus arteriosus (PTA), and bicuspid aortic valve disease (BAV) [81]. A favorable CHD prognosis and reduced mortality is associated with early and correct diagnosis. However, the standard diagnostic method to detect CHD usually includes echocardiography and has an efficiency rate up to 35%, which is considerably low [82]. Additionally, some CHDs are diagnosed only after birth, during infancy, or even during adulthood. During pregnancy, several cardiogenesis-associated ncRNAs are potentially secreted by the fetus and enter into the maternal bloodstream, where they can be detected by blood analysis and used as biomarkers for CHD [83]. Multiple studies have also linked specific CHDs with an altered miRNA expression profile. In particular, children with ASD present a significant upregulation of let-7a and miR-486 and, although not statistically significant, an increase in hsa-let-7b levels when compared with healthy children [84]. Interestingly, mothers of ASD children express a similar miRNA expression pattern. In fact, because let-7a expression levels positively correlated between mothers and their offspring [84], its levels in the mother could predict ASD in children with a sensitivity of 82% and a specificity of 91%, indicating a significant diagnostic value of this miRNA in detecting ASD in children [84].

Bicuspid aortic valve disease (BAV), the most common anomaly of the human heart, results from an anomaly during valvulogenesis that is characterized by no splitting between two adjacent cusps, and it is frequently accompanied by dilation of the ascending aorta [85]. The most prominent miRNAs in adult BAV patients seem to be miR-122, miR-130a, and miR-486, and miR-718, with the latter being altered in BAV patients and inversely correlating with the diameter of the ascending aorta and, therefore, suggested as a potential biomarker of aortic dilation [86].

Overall, CHDs can be distinguished from non-CHDs through specific alterations in miRNA expression patterns [87]. Namely, miR-125b-2-3p, miR-1284, miR-142-5p, miR145-3p, miR-4426, miR-4666a-3p, and miR-4681 are downregulated, while miR-1275, miR-3664-3p, and miR4796-3p are upregulated in women pregnant with a CHD child compared with women pregnant with a non-CHD child [87]. Notably, such differences are no longer observed 24 h after delivery, supporting the hypothesis that the above miRNAs are pregnancy-related. ROC analysis demonstrated miR-142-5p to have the highest diagnostic accuracy with an AUC of 0.804 [87]. Indeed, the combination of the top four miRNAs (miR-142-5p, miR-1275, miR-4666a-3p, and miR-3664-3p) improved the accuracy in discriminating CHD from controls [87]. Interestingly, specific miRNAs can also be related to a particular CHD phenotype. For example, VSD revealed changes in miR-142-5p, miR-1275, miR-4666a-3p, and miR-3664-3p; TOF related to dysregulation of miR142-5p, miR-4666a-3p, and miR-3664-3p, while both SV and PTA were linked to miR-142-5p and miR-3664-3p differential expression [87].

A relatively small study, including 22 pregnant CHD-positive and 17 CHD-free controls, suggested miR-99a to be upregulated in the peripheral blood of the CHD-positive pregnant women when compared with the control group [88]. However, no diagnostic value was determined for this miRNA.

Yu et al. also suggested that a different group of miRNAs could identify CHDs such as ventricular septal defect (VSD), atrial septal defect (ASD), and tetralogy of fallot (TOF) [83]. The group composed of miR-19b, miR-22, miR-29c, and miR-375 is upregulated in maternal serum of CHD fetuses when compared with maternal serum of non-CHD fetuses [83]. When miRNAs were clustered according to the disease phenotype, all miRNAs were significantly upregulated in TOF, whereas only miR-19b and miR-29c were significantly increased in VSD and miR-19b, miR-29c, and miR-375 were increased in ASD [83]. Although all the miRNAs demonstrated a significant discriminatory value, and thus could be used as a disease biomarker for the detection of fetal CHD, the combination of all four miRNAs proved to be a more efficient diagnostic strategy.

Regarding lncRNAs in CHDs, HOTAIR was found to be upregulated in both myocardial tissue and plasma samples of ASD, VSD, and patent ductus arteriosus (PDA) patients when compared with healthy subjects, and thus was indicated as a potential biomarker for CHD [89]. HOTAIR seems to work through recruitment of PRC2 and inducing epigenetic modifications during embryonic heart development [89].

From a microarray-based strategy, 17,603 lncRNAs were found to be differentially present in the plasma of fetal CHD pregnant women [82]. From these, 3694 lncRNAs were significantly upregulated and 3919 were downregulated. Subsquently, this subgroup was subjected to gene ontology (GO) analysis to describe their association with specific biological processes, cellular components, and molecular functions through. GO analysis recognized 26 CHD-related lncRNAs, among which four were differentially altered in VSD, as well as another four in TOF, and two others were significantly different in ASD [82]. Overall, pregnant women with fetal CHD revealed alterations in ENST00000436681, ENST00000422826, AA584040, AA709223, and BX478947, when compared with the control group [82], all of them showing a moderated discriminating effect; therefore, it was suggested as potential lncRNA biomarkers to predict fetal CHD.

More recently, the possible value of circRNAs in the diagnosis of CHD has also been addressed. A study including 40 children with CHD (namely VSD and ASD) and 40 healthy children identified 10 upregulated and 157 downregulated circRNAs in the plasma of the CHD group. Validation by qPCR demonstrated three circRNAs, hsa_circRNA_004183, hsa_circRNA_079265, and hsa_circRNA_105039, to be significantly decreased in the affected group [90]. Hsa_circRNA_105039 presented the best diagnostic value with a sensitivity of 80%, a specificity of 100%, and AUC of 0.907, but altogether, the three circRNAs had an AUC of 0.965, suggesting a combinatorial approach to be effective in the diagnosis of CHD [90].

## 6. Right Ventricle Dysfunction

The right ventricle (RV) is responsible for pumping venous blood into the pulmonary vascular bed on the way to the LV, thereby contributing to left ventricular filling and cardiopulmonary function. Its thin walls and the direct connection with low impedance pulmonary circulation make the RV more sensitive to pressure than to volume overload [91]. Pulmonary hypertension (PH), pulmonary embolism, RV infarction, and cardiomyopathies are among the main causes of RV dysfunction and, consequently, failure [92]. Despite its critical contribution to heart function, the RV has been somewhat neglected, with only a few studies having specifically focused on RV failure. The limited knowledge of RV biology and function contributes to the poor prognosis of RV failure (RVF) patients, with both treatment and diagnosis of RVF remaining challenging. Conventional diagnostic approaches are based on ECG, 3D-echocardiography and strain imaging, magnetic resonance imaging, and blood marker analysis such as lactate and BNP levels [93]. Although ncRNAs also recently started receiving attention in the RV field, there is still a scarce number of studies reporting on ncRNAs as biomarkers on RVF.

Arrhythmogenic cardiomyopathy (ACM) is a life-threating genetic disease where RV myocardial tissue is replaced by fibro-fatty tissue, leading to arrhythmias and, eventually, HF [94]. ACM is particularly challenging to detect because of variance expression and penetrance of the mutated gene, and as a consequence, genetics tests can result in inconclusive diagnostics [94]. miRNA profiling studies revealed miR-320a to be downregulated in ACM patients, when compared with healthy controls [94]. Intermediate values of sensitivity and specificity obtained for miR-320a were increased when combined with EGC analysis, thus improving its diagnostic potential [94]. Despite these promising results, so far, no correlation between miR-320a levels and disease severity has been found [94].

PH is a multifactorial disease, with several subgroups being characterized by an increase in RV afterload that can precede (right) HF and, eventually, death. The first report on PH-associated circulating miRNAs analyzed plasma samples from PH patients undergoing right heart catheterization [95]. MiRNAs such as miR-1, miR-26a, miR-29c, miR-34b, miR-451, and miR-1246 were found to be downregulated, whereas miR-130a, miR-133b, miR-191, miR-204, and miR-208b were profoundly upregulated in PH subjects when compared with control subjects. From all these, only miR-208b and miR-130 could be correlated with PH severity [95].

Another similar study revealed miR-451 as being significantly decreased in PH patients compared with non-PH patients (individuals diagnosed with other conditions) [96]. Although the diagnostic value of miR-451 was as moderate as the conventional Doppler-echo, combing both improved the diagnostic value of miR-451 in PH [96]. More recently, miR-424 was also linked to PH and found to be upregulated in plasma of PH patients [97], with patients with severe PH clearly displaying higher miR-424 levels when compared with individuals with less severe PH [97]. As miR-424 inversely correlates with cardiac output parameters in PH patients, it has been suggested has a potential marker of PH [97].

## 7. Pathological Reverse Remodeling

Cardiac pathological remodeling associated with the above-mentioned different cardiovascular diseases can lead to HF and, eventually, death. The severity of cardiac pathological remodeling frequently predicts treatment outcome and patient response [98]. In light of the current findings, such remodeling can be reversed and result in improved of cardiac function and, consequently, better patient prognosis and survival [99,100,101]. Reverse remodeling (RR) can be defined by any functional, structural, cellular, and/or molecular changes, resulting in improved heart function following pathological remodeling [99]. Factors contributing to the process include both pharmacological angiotensin-converting enzyme inhibitors, beta-blockers, and mechanical therapy such as cardiac resynchronization therapy (CRT), PCI, left ventricle assist device implantation (LVAD), and AVR surgery, among others [99]. Unfortunately, a fraction of patients undergoing cardiac therapy do not develop a favorable clinical response; do not show improved cardiac function; and, in some cases, cardiac condition is worsened after treatment [99,102]. Such patients, with incomplete reverse remodeling, are classified as non-responders, while patients with improved cardiac function and complete reverse remodeling are categorized as responders [99].

Understanding the features of cardiac pathological remodeling and the cardiac response to therapy has become a major objective towards better care of CVD patients. In line, biomarkers, and more specifically ncRNAs, are claiming a greater role towards establishing reverse remodeling and predicting patient outcome upon specific therapies.

Presently, the only therapy that consistently increases survival of AS patients is AVR surgery. However, the persistence of hypertrophy after AVR surgery is a main limiting factor for patient survival and positive outcomes. Circulating miR-133a levels prior to AVR surgery are a positive predictor of LV mass normalization and subsequent decrease of cardiac hypertrophy up to one year after surgery. When combined with other clinical parameters, miR-133a yielded an AUC of 0.89 [103] and is currently considered one of the most promising biomarkers to predict LV normalization in AS patients after AVR [103].

A negatively patient outcome after mechanical therapy such as coronary artery bypass graft or AVR due to CAD and AS, respectively, is associated with high levels of serum miR-423-3p [104]. Although this is specifically significant in patients with unstable angina, when compared with individuals with stable angina or AS, the reason that miR-423-3p is particularly elevated in these patients is inconclusive [104].

Another study collectively addressed miRNA expression in the Pro-BNP Outpatient Tailored CHF Therapy (PROTECT) cohort to predict left ventricle reverse remodeling (LVRR) based on miRNAs that have been involved in human HF phenotypes or known to influence common HF signaling pathways [105]. Only 41% of the participants exhibited complete RR, considered as more than 15% reduction in LV end-systolic volume index [105]. Not only PCA analysis identified miR-423-5p and miR-212-5p as dysregulated in the plasma of systolic HF patients, they were also associated with LVRR and shared 14 target genes related to HF [105]. Together with the fact that miR-423-5p and miR-212-5p are also upregulated in myocardium tissue of end-stage HF in mice, these findings support the prospective role of miRNAs in discriminating LVRR [105].

Aside from the merit of circulating miRNAs, lncRNAs such as LIPCAR, H19, ANRIL, and MHRT have also emerged as potential biomarkers for LVRR. For example, higher plasma levels of LIPCAR and H19 in CAD patients are associated with chronic HF [106]. In HF patients, higher levels of LIPCAR are associated with a higher risk of hospitalization and mortality due to HF when compared with HF patients with lower LIPCAR levels [107]. Similarly, plasma MHRT is significantly lower in patients with HF and is able to distinguish HF from healthy subjects by presenting an AUC of 0.925 [108]. Among HF patients, lower levels of plasma MHRT significantly correlate with a lower survival rate when compared with HF patients displaying higher MHRT levels.

As previously mentioned, one treatment for CAD is coronary intervention by the use of a stent; however, 12% of patients with stent therapy showed angiographic restenosis, in-sent restenosis (ISR) [109]. ISR is defined as a “narrowing of a coronary artery at the stented segment”, and is associated with increased plasma levels of ANRIL, when compared with CAD patients without restenosis [110]. To note, ANRIL was found as an independent risk factor for ISR incidence, with a modest diagnostic value demonstrated by an AUC of 0.749. In addition, ANRIL demonstrated a specificity of 75% and sensitivity of 68.4% after a cutoff value of 1.34 [110].

Finally, plasma circRNA MICRA was found to be a strong predictor of LV dysfunction 3 to 4 months after an AMI episode, and lower MICRA levels in MI patients are at higher risk of LV dysfunction [80].

## 8. Conclusions

**Figure 1:**
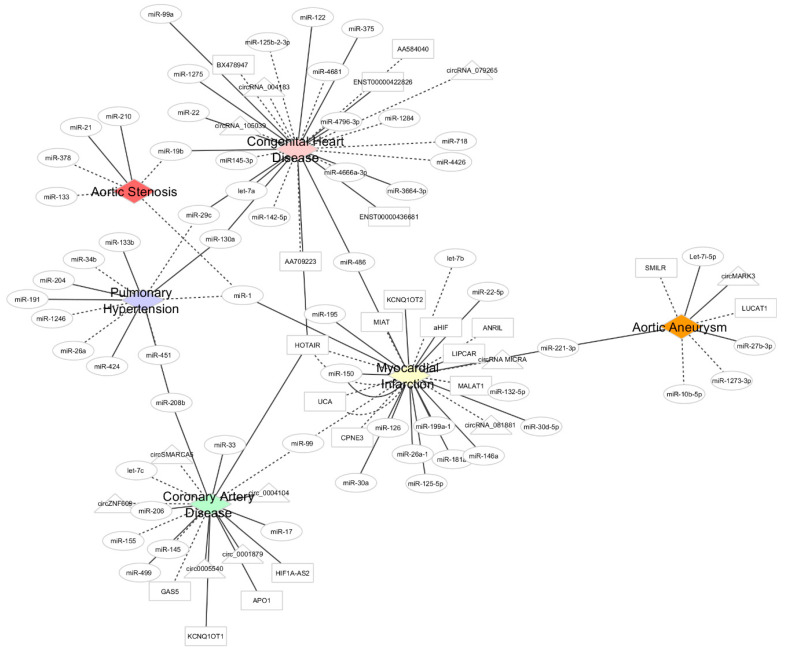
Illustrative cytoscape network combining the studies reported within this review. Aortic valve stenosis (red), congenital heart disease (pink), coronary artery disease (green), myocardial infarction (yellow), aortic aneurism (orange), and pulmonary hypertension (blue) are the five central nodes (diamond shape) and specific associations to ncRNAs are denoted by edges. The different ncRNAs are represented by different edges shapes: miRNAs are in circular shape, lncRNAs are in rectangular shape, and circRNAs are in triangular shape. Dashed lines represent upregulated plasma ncRNAs, while solid lines represent downregulated ncRNAs in plasma.

Misdiagnosed CVDs have severe consequences on a patient’s life quality and associated survival rate. The use of standard biomarkers for CVDs as the quantitative assessment of circulating cardiac troponins and natriuretic peptides levels it is still not specific and neither sensitive enough for early detection. Moreover, the complexity of many biological and molecular mechanisms observed on the different CVDs makes the use of a single biomarker insufficient for a correct diagnosis. Currently, circulating ncRNAs display great potential as biomarkers owing to their high abundance and stability in the blood, and ncRNAs can provide a valuable measure of heart health condition. Therefore, ncRNAs have been portrayed as potential non-invasive tools and might help on CVDs’ diagnosis and prognosis, allowing tailored healthcare to each individual and a reduction of the associated societal and economic burden. However, the expression profile of ncRNAs could be influenced by age, sex, and medical drugs, which might add more complexity to our understanding of ncRNAs in CVDs. Furthermore, up to date, only a scarce number of works reported a higher individual diagnostic value of ncRNAs when compared with standard CVD markers; however, frequently, this value is improved when a set of ncRNAs is used or combined with standard CVD markers. The majority of the studies are characterized by a smaller sample size, with some possible cofounders associated (region, ethnicity, age, social status), and a short-term follow-up thwarts the hunting for the ideal biomarker. Interestingly, some ncRNAs such as muscle specific miR-1 and lncRNA HOTAIR are reported in several diseases (Table 1), suggesting a feature of a general CVD biomarker, while others appear to have disease-specificity (Figure 1 and Supplementary Table S1). New findings on ncRNAs and their role as blood-based biomarkers are reported frequently, such as the study of Viereck, who performed an extensive review on this topic [111]. Despite the vast amount of data on CVDs and plasma ncRNAs, their inclusion in medical guidelines is still far off in the upcoming years. Thus, we recommend researchers who aim to validate potential biomarkers in big cohorts to rely on the candidates derived from studies that include the largest sample sizes, on biomarkers that have the high sensitivity and specificity, and on biomarkers that show a significant correlation with cardiac parameters associated with the specific cardiovascular disease.

## Figures and Tables

**Table 1:** List of non-coding RNAs (miRNAs, lncRNAs, and circRNAs) involved in multiple cardiovascular diseases. AS, aortic valve stenosis; MI, myocardial infarction; PH, pulmonary hypertension; TOF, tetralogy of fallot; VSD, ventricular septal defect; ASD, atrial septal defect; PDA, patent ductus arteriosus; BAV, bicuspid aortic valve disease; CAD, coronary artery disease. Arrows indicate the direction of miRNA variation: ↓ and ↑ correspond to a under-expression and over-expression of the miR.

NcRNA	Disease	Variation	Reference
miR-1	AS	↓	[29]
	MI	↑	[68]
	PH	↓	[95]
miR-150	MI	↑	[69]
	MI	↑	[72]
miR-19b	TOF	↑	[83]
	VSD	↑	[83]
	ASD	↑	[83]
	AS	↓	[34]
miR-208b	CAD	↑	[52]
	PH	↑	[95]
miR-29c	TOF	↑	[83]
	ASD	↑	[83]
	PH	↓	[95]
	VSD	↑	[83]
miR-375	TOF	↑	[83]
	ASD	↑	[83]
miR-486	MI	↑	[72]
	ASD	↑	[84]
	BAV	↑	[86]
miR-99	CAD	↓	[62]
	MI	↓	[62]
NcRNA	Disease	Variation	Reference
HOTAIR	CAD	↑	[56]
	ASD	↑	[89]
	VSD	↑	[89]
	PDA	↑	[89]
	MI	↓	[77]
	MI	↓	[75]
KCNQ1OT1	CAD	↑	[55]
	MI	↑	[77]
UCA	MI	↓	[76]
	MI	↓	[77]
NcRNA	Disease	Variation	Reference
circRNA_004183	ASD	↓	[90]
	VSD	↓	[90]
circRNA_079265	ASD	↓	[90]
	VSD	↓	[90]
circRNA_105039	ASD	↓	[90]
	VSD	↓	[90]
circRNA MICRA	MI	↓	[79]
circRNA MICRA	MI	↓	[80]

## Supplementary Materials

Supplementary materials can be found at https://www.mdpi.com/1422-0067/21/23/9285/s1.
