# Supplementary material for: Non-Coding RNAs as Blood-Based Biomarkers in Cardiovascular Disease
Source: Int J Mol Sci. 2020 Dec 5;21(23):9285. doi: 10.3390/ijms21239285 (PMC7730567; doi:10.3390/ijms21239285)
Supplement: Supplementary file 1 [file ijms-21-09285-s001.pdf]

Supplementary Table 1. Non-coding RNA (miRNAs, lncRNAs, and circRNAs) variation in several cardiovascular diseases**.** TAA, thoracic aortic aneurysm; AAA, abdominal aortic aneurysm; CHD, congenital heart defect.

ncRNA	Disease	Variation	Reference
let-7a	ASD	↑	[85]
let-7b	MI	↓	[66]
let-7c	CAD	↓	[52]
Let-7i-5p	AAA	↑	[47]
miR-1	AS	↓	[29]
	MI	↑	[69]
	PH	↓	[96]
miR-10b-5p	AAA	↓	[47]
miR-122	BAV	↑	[87]
miR-1246	PH	↓	[96]
miR-125-5p	MI	↑	[71]
miR-125b-2-3p	CHD	↓	[88]
miR-126	MI	↑	[68]
miR-1273-3p	AAAD	↓	[49]
miR-1275	CHD	↑	[88]
miR-1284	CHD	↓	[88]
miR-130a	BAV	↑	[87]
miR-130a	PH	↑	[96]
miR-132-5p	MI	↓	[70]
miR-133	AS	↓	[29]
miR-133b	PH	↑	[96]
miR-142-5p	CHD	↓	[88]
miR-145	CAD	↓	[52]
miR145-3p	CHD	↓	[88]
miR-146a	MI	↑	[67]
miR-150	MI	↑	[70]
	MI	↑	[73]
miR-155	CAD	↓	[52]
miR-17	CAD	↑	[51]
miR-181a	MI	↑	[64]
miR-191	PH	↑	[96]
miR-195	MI	↑	[66]
miR-199a-1	MI	↑	[67]
miR-19b	TOF	↑	[84]
	VSD	↑	[84]
	ASD	↑	[84]
	AS	↓	[34]
miR-204	PH	↑	[96]
miR-206	CAD	↑	[55]
miR-208b	CAD	↑	[53]
	PH	↑	[96]
miR-21	AS	↑	[32]
miR-210	AS	↑	[30]
miR-22	TOF	↑	[84]
miR-221-3p	MI	↑	[72]
	AAA	↑	[46]

miR-22-5p	MI	↑	[70]
miR-26a	PH	↓	[96]
miR-26a-1	MI	↑	[67]
miR-27b-3p	AAA	↑	[46]
miR-29c	TOF	↑	[84]
	ASD	↑	[84]
	PH	↓	[96]
	VSD	↑	[84]
miR-30a	MI	↑	[66]
miR-30d-5p	MI	↑	[71]
miR-33	CAD	↑	[54]
miR-34b	PH	↓	[96]
miR-3664-3p	CHD	↑	[88]
miR-375	TOF	↑	[84]
	ASD	↑	[84]
miR-378	AS	↓	[29]
miR-424	PH	↑	[98]
miR-4426	CHD	↓	[88]
miR-451	PH	↓	[96]
miR-451	PH	↓	[97]
miR-4666a-3p	CHD	↓	[88]
miR-4681	CHD	↓	[88]
miR-4796-3p	CHD	↑	[88]
miR-486	MI	↑	[73]
	ASD	↑	[85]
	BAV	↑	[87]
miR-499	CAD	↑	[53]
miR-718	BAV	↓	[87]
miR-99	CAD	↓	[63]
	MI	↓	[63]
miR-99a	CHD	↑	[89]

ncRNA	Disease	Variation	Reference
AA584040	CHD	↓	[83]
AA709223	CHD	↓	[83]
aHIF	MI	↑	[78]
ANRIL	MI	↓	[78]
APO1	CAD	↑	[56]
BX478947	CHD	↓	[83]
CPNE3	MI	↓	[78]
ENST00000422826	CHD	↑	[83]
ENST00000436681	CHD	↑	[83]
GAS5	CAD	↓	[58]
HIF1A-AS2	CAD	↑	[56]
HOTAIR	CAD	↑	[57]
	ASD	↑	[90]
	VSD	↑	[90]
	PDA	↑	[90]
	MI	↓	[78]
	MI	↓	[76]
KCNQ1OT1	CAD	↑	[56]
	MI	↑	[78]
LIPCAR	MI	↑	[78]

LUCAT1	TAA	↓	[48]
MALAT1	MI	↓	[78]
MIAT	MI	↓	[78]
SMILR	TAA	↓	[48]
UCA	MI	↓	[77]
	MI	↓	[78]

ncRNA	Disease	Variation	Reference
circ_0001879	CAD	↑	[62]
circ_0004104	CAD	↑	[62]
circ0005540	CAD	↑	[60]
circRNA_004183	ASD	↓	[91]
	VSD	↓	[91]
circRNA_079265	ASD	↓	[91]
	VSD	↓	[91]
circRNA_081881	MI	↓	[79]
circRNA_105039	ASD	↓	[91]
	VSD	↓	[91]
circMARK3	AAAD	↑	[49]
circRNA MICRA	MI	↓	[80,81]
circSMARCA5	CAD	↓	[59]
circZNF609	CAD	↓	[61]
